# Synthesis of new pyrazolone and pyrazole-based adamantyl chalcones and antimicrobial activity

**DOI:** 10.1042/BSR20201950

**Published:** 2020-09-24

**Authors:** Rawan Al-Saheb, Sami Makharza, Feras Al-battah, Rajab Abu-El-Halawa, Tawfeq Kaimari, Omar S. Abu Abed

**Affiliations:** 1 Department of Chemistry, Faculty of Science, Hebron University, Hebron, Palestine; 2 College of Pharmacy and Medical Sciences, Hebron University, Hebron, Palestine; 3 Biology and Biotechnology Department, Faculty of Arts and Sciences, Arab American University in Palestine (AAUP), Jenin, Palestine; 4 Department of Chemistry, Faculty of Science, University of Al al-Bayt, Al-Mafraq, Jordan; 5 Department of Health Sciences, Faculty of Graduate Studies, Arab American University in Palestine (AAUP), Ramallah, Palestine

**Keywords:** Adamantyl Chalcones, antibacterial activity, Chalcone, Pyrazole, Pyrazolone

## Abstract

Chalcones and their derivatives are becoming increasingly popular due to their various pharmacological effects. Chalcone molecules may be extracted from natural resources, entirely synthesised, or biosynthesised by modifying the natural ones. In the present study, five pyrazole-based adamantyl heterocyclic compounds were synthesised by condensation of 1-adamantyl chalcone with substituted phenylhydrazine. The products were characterised by using ¹H NMR, ¹³C NMR and FT-IR spectroscopy. The microbiological activity of these compounds was investigated against bacteria and fungi. The new compounds showed good to moderate activity against the microbial species used for screening. All developed molecules showed antibacterial activity against Gram-negative and Gram-positive. These molecules showed antifungal activities against *Fusarium oxysporum* fungus and in a dose-dependent manner, apart from RS-1 molecules which showed compromised antifungal activity and even at a high dose.

## Introduction

Chalcone is a core structure of a wide variety of naturally originated, synthetic and semisynthetic molecules [1]. In nature, a plethora of foods and plants are considered rich sources of chalcone and its derivatives, e.g. tea, vegetables, fruits, soy and spices [2]. In the past decades, the chalcones have attracted considerable attention of researchers due to their enormous and unique biological and pharmacological activities [3]. The most significant therapeutic applications of the chalcones are their antibacterial [4,5], antifungal [6], antimicrobial [7], anticancer [8], antioxidant [9] and anti-inflammatory [10] activities. The discovery of chalcone compounds provides an outstanding case history of modern drug development and also reveals their promising biological activities as a result of the structural modification of prototype drug molecules [3,11].

Chalcone (E)-1,3-diphenyl-2-propen-1-one is an open-chain flavonoid found in several natural sources [12] or prepared by condensation of phenylmethyl ketone with phenyl aldehyde in the presence of suitable condensing agents [13,14]. It undergoes a chemical reaction that leads to the preparation of heterocyclic compounds having pharmacological activities. Therefore, the synthesis of modified and customised chalcones is becoming more popular than ever before. There are approximately 100000 chalcone-derived molecules can be found in the literature [11], and amongst them, more than 2000 molecule have shown biological activity as reported in PubChem (accessed April 2020). Although the mechanism of the biological actions of chalcones is still not entirely revealed, various pharmacological uses of chalcones are attributed to the existence of an α, β-unsaturated ketone system [3].

Most of chalcone molecules are categorised within two main classes; they are either simple chalcone structure or composite of the core of the unsaturated ketone backbone with other added molecules to enhance their biological properties [11]. Although the novel pharmacological effects are usually related to typical chalcone, they are also associated with a wide range of natural or pioneered hybrid chalcone molecules which obviously lack the enone group, e.g. heterocyclic chalcones [15]. Chalcones have an innate ability to make several heterocycles in its structure depending on the different factors, e.g. reaction conditions and the position of hydrogen atoms [16]. The heterocyclic compounds in the chalcones; such as pyrazoles, pyrazoline, or pyridine, exhibit biological, antitumour, antimicrobial and anti-inflammatory activities [17–21]. Hence, the synthesis of chalcone derivatives is always a great challenge.

Different modifications may be needed to have potential effects in overcoming multidrug-resistant bacterial and fungal infections. Bacterial resistance to existing antibiotics is approaching alarming levels, largely because of the wide misuse and poor patient compliance. Together with the decline in the rate of discovery of new antibiotics, a major crisis in healthcare for infectious diseases is yet to evolve [22,23]. Therefore, there is a serious need to investigate novel classes of antimicrobial agents, with appropriate therapeutic, toxicological, and pharmacokinetic properties [24]. Chalcones and their derivatives exhibit potent antioxidant and antibiotic properties, which serves as plants’ defence mechanism against reactive oxygen species and microbial infections. Therefore, chalcones and chalcones derivatives are increasingly considered for the development of novel therapies to tackle oxidative stress-driven pathologies such as infectious and inflammatory diseases [25].

Anderson and Kaimari, 2013 modulated the general chalcone structure by the replacement of the aryl ring A with a hydrocarbon moiety (adamantyl group) and the aryl ring B with heteroaryl moieties (pyridine) [26]*.* In the general formula of 1-adamantyl chalcones, adamantly and pyridine group are linked by a three-carbon α, β-unsaturated carbonyl system. The structure has lipophilicity characters; adamantyl group ensures favourable condition for its transport through a biological membrane [26,27], and the presence of pyridine and α, β-unsaturated groups improves their biological activities [28]. Adamantyl chalcones were prepared by condensation of 1-adamantyl methyl ketone with pyridine-2-carboxaldehyde in the presence of suitable condensing agent KOH, then undergoes a variety of chemical reactions leading to many heterocyclic compounds. In the present study, pyrazolone and pyrazole derivatives of adamantyl chalcone compounds were prepared, wherein the chalcone moiety is an intermediate to new compounds with therapeutic value.

Pyrazole is an organic heterocyclic compound characterised by a 5-membered ring containing three carbon atoms and two adjacent nitrogen atoms with the formula C_3_H_4_N_2_. It is a weak base with p*K*_b_ 11.5. One of the nitrogen atoms is neutral in nature, and the other is basic [18]. Due to its planar conjugated ring structure, pyrazole is an aromatic molecule with six delocalised π-electrons. The aromatic nature arises from the unshared pair of electrons on the nitrogen (–NH) and the four π-electrons in the ring [18]. The partially reduced forms of pyrazole are named pyrazolines. These derivatives play an essential role in heterocyclic compounds history and possess considerable biological activities; thus, making them important pharmacophores for carrying out further drug development research.

Several methods have been developed for the preparation of substituted pyrazole, one of the most important methods is the reaction between α, β-unsaturated chalcone with hydrazine derivatives. Several catalysts have been developed for the preparation of these heterocycles, including sodium acetate/acetic acid aqueous solution under ultrasound irradiation [29], hot acetic acid solution [19,30], potassium hydroxide/ethanol under reflux [15], sodium acetate/ethanol under reflux [31], methanoic acid/ethanol under reflux [32], glacial acetic acid under ultrasonic irradiation [33], phosphotungstic acid (PTA) catalyst/ethanol [34], and microwave irradiation [35].

The present study aims to pioneer five new adamantyl-based chalcones modified with pyrazole and pyrazolone as potential antibacterial and antifungal agents with superior chemical properties to overcome some of the biological and pharmacological properties. To our knowledge, this is the first study that reports the development of pyrazole and pyrazoline adamantyl chalcones.

## Materials and methods

1-Adamantyl methyl ketone (99%), pyridine-2-carboxaldehyde (99%), potassium hydroxide (90%), 2,4-dinitrophenylhydrazine (97%), phenylhydrazine hydrochloride (99%), 2-chlorophenyl hydrazine hydrochloride (97%), 4-methoxy-phenyl hydrazine hydrochloride (98%), *o*-tolyl hydrazine hydrochloride (97%), concentrated sulfuric acid, glacial acetic acid, sodium acetate, ethyl acetate, hexane, methanol, and ethanol were obtained from Sigma–Aldrich.

Gram-negative bacteria species *Pseudomonas aeruginosa (ATCC/27853), Klebsiella pneumonia, Salmonella typhimurium* and *Escherichia coli (ATCC/25922)*, and Gram-positive bacteria species *Bacillus subtilis* and *Staphylococcus aureus (ATCC/25923)* were obtained from Agricultural Biological Laboratory. Gram-negative bacteria (*Meropenem, Gentamycin)* and Gram-positive bacteria antibiotics *(Meropenem, Ampicillin)* were used as antibiotic standards, normal saline 0.9%, Mueller–Hinton agar media (MHA), Potato dextrose agar (PDA), chloramphenicol (99%), *Fusarium oxysporum* fungus were obtained from Agricultural Laboratory.

## Instruments

^1^H-NMR apparatus was used to determine the proton spectra of compounds. The spectra were recorded in a deuterated solvent CDCl_3_ in 400 MHz at 25°C or by DMSO-d6 solvent in 300 MHz at 21°C. Chemical shifts (δ) are given in parts per million (ppm) downfield relative to tetramethylsilane, TMS. The ^13^C-NMR apparatus utilised to identify the carbon atoms in compounds, the spectra recorded in a deuterated solvent CDCl_3_ in 400 MHz at 25°C. The chemical shift reference standard for ^13^C is the carbons in TMS, whose chemical shift is considered to be 0.0 ppm.

The FT-IR spectroscopy provides detailed information about the structural changes, KBr thin disc was used to determine IR spectrum for the solid compounds in the region (4000–400 cm^−1^). Thin-layer chromatography (TLC) plastic sheets silica gel, 20 × 20 cm, layer thickness 0.2 mm, was eluted with an ethyl acetate/hexane mixture, the spots were detected by UV light which monitored the progress of the reaction. The open capillary was used to determine the melting points of the compound use Electro-Thermal Stuart SMP3 advanced melting point apparatus.

## Experimental

### Synthesis of adamantyl chalcone

A total of 0.0028 mol of 1-adamantyl methyl ketone was added to an ethanolic solution (0.0028 mol of KOH in 40 ml ethanol 96%) and stirred for 15 min. A total of 0.0028 mol of pyridine-2-carboxaldehyde was added dropwise to the solution and stirred at room temperature for 48 h. The adamantly chalcone was obtained according to the previously published method [26]*.* Briefly, the reaction mixture was poured into ice water until solid yellow crystals were formed. Then, the system was filtered and washed with a mixture of ethyl acetate:hexane (2:5) several times and dried at room temperature. The progress of reaction and the extent of purity was monitored throughout the process by utilising TLC.

### Synthesis of pyrazolines

**Figure 1:**
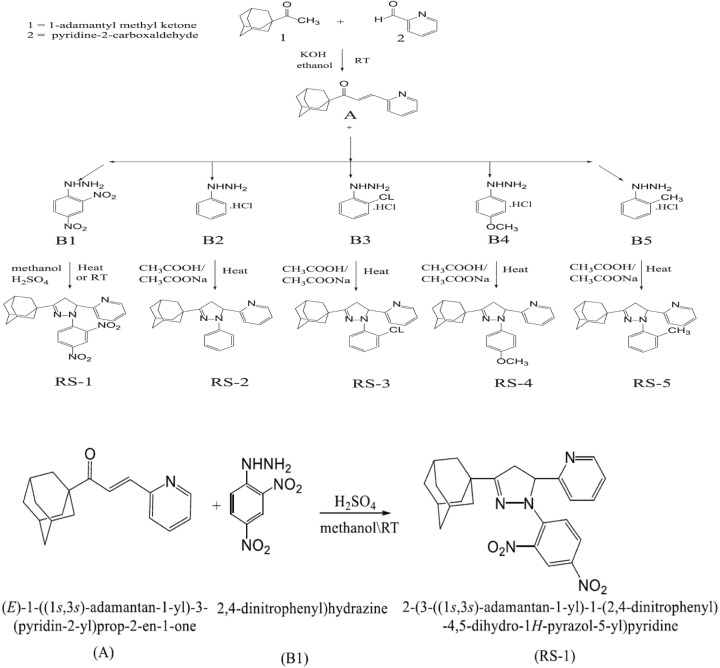
General scheme reactions for the synthesis of five pyrazoline adamantyl compounds

The 1-adamantyl-3-pyridyl-prop-2-en-1-one (A) reacts with substituted phenylhydrazine (B 1-5) to form trisubstituted 4,5-dihydropyrazoles (RS 1-5) as shown in Figure 1.

### Synthesis of *RS-1* compound

**Figure 2:**
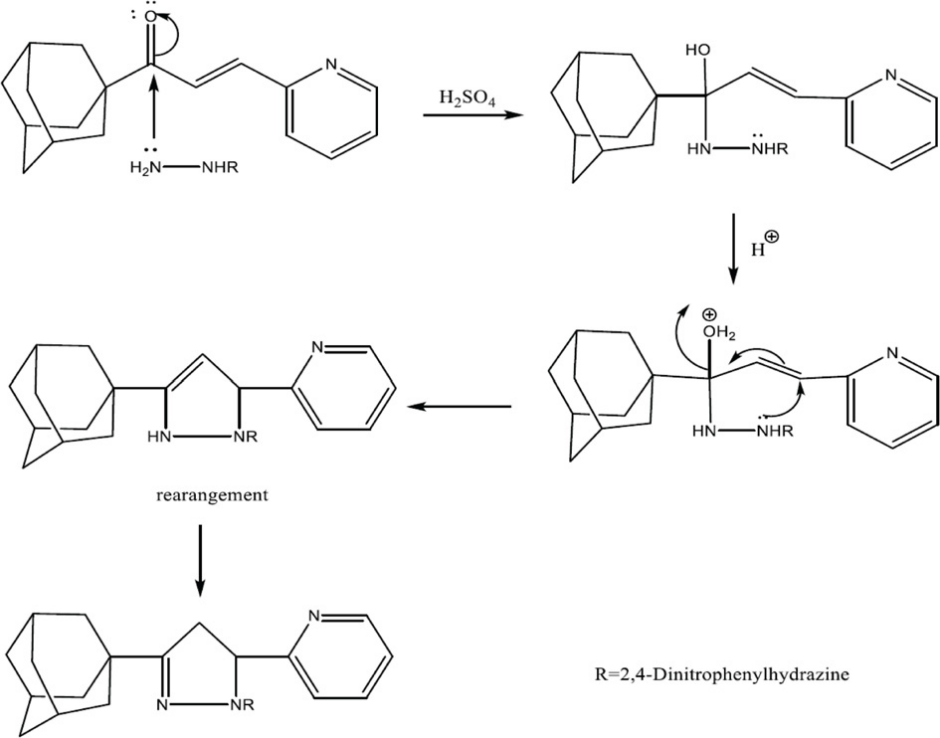
Detailed scheme reaction for the synthesis of RS-1 pyrazoline adamantyl compounds

A total of 0.5 mmole of adamantly chalcone was dissolved in 3 ml of methanol under stirring, 0.5 mmol 2,4-dinitrophenyl hydrazine (compound B1) was dissolved in 5 ml of methanol and 0.3 ml of concentrated sulfuric acid, the adamantly chalcone was added to compound B1 solution under stirring for 48 h at room temperature, Figure 2.

### Synthesis of *RS-2, RS-3, RS-4*, and *RS-5* compounds

**Figure 3:**
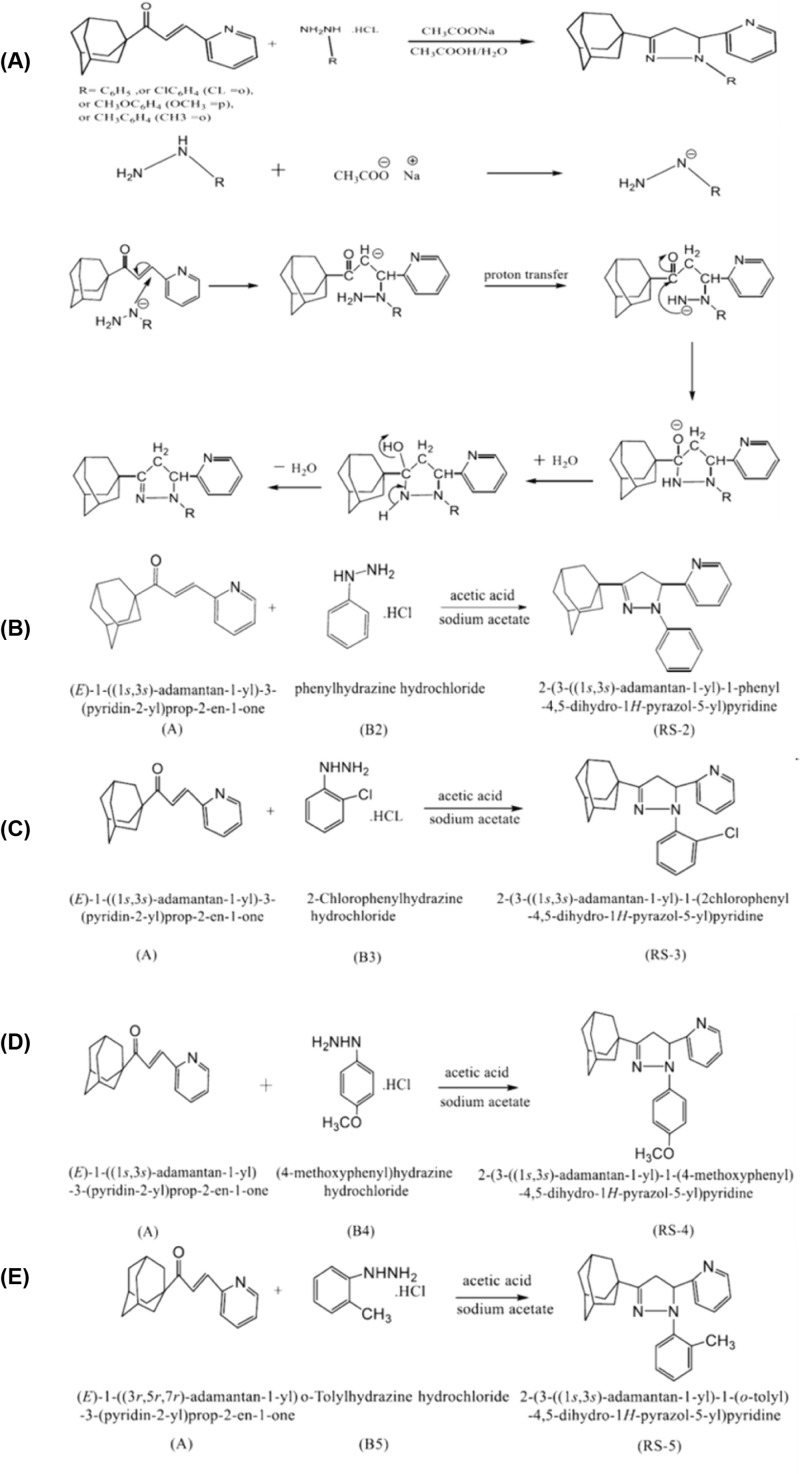
Detailed scheme reaction for the synthesis of RS2-5 pyrazoline adamantyl compounds (**A**) General reaction between admantyl chalcone and phenylhydrazine hydrochloride derivatives. The other schemes represent the reaction of admantyl chalcone with (**B**) phynelhydralzine hydrochloride to produce RS-2, (**C**) 2-chlorophenylhydrazine hydrochloride for RS-3, (**D**) (4-methoxyphenyl)hydrazine hydrochloride for RS-4, and (**E**) *o*-tolylhydrazine hydrochloride for RS-5.

In separate experiments, 0.75 mmol mixture of adamantly chalcone, 0.75 mmol phenylhydrazine hydrochloride derivatives (B2-5) and 0.15 mmol sodium acetate were dissolved in acetic acid aqueous solution (6 ml, HOAc/H_2_O = 2/1, v/v), as illustrated in Figure 3. The mixture was stirred and heated at 80°C for 48 h. The reaction mixture was poured into crushed ice; the solution was evaporated in the rotovap in order to obtain the pyrazoline product.

### Antibacterial activity by agar diffusion method

The prepared compounds were screened for their minimum inhibitory concentratio (MIC) activity by agar diffusion method [36]. Sterile Petri MHA media was cultured with bacteria, then placed the prepared antibiotic discs on the inoculated agar plate, the antibiotic discs were prepared approximately 6 mm in diameter. The discs autoclaved at (12 lbs) pressure for 15 min. Sterile discs were placed in Petri dishes approximately 5 mm apart. A fixed volume of 20 μl was loaded on each disc one by one using a pipette. Then the Petri dishes incubated at 37°C overnight. After the incubation period, the zone of inhibition was measured for each of the antibiotic discs, and the lowest concentration required to arrest the growth of bacteria was calculated.

### Antibacterial activity by disc diffusion method

Gram-negative and Gram-positive bacteria species were used as antibacterial test strains. The compounds ***RS 1-5*** were screened at the concentration (500 μg/ml) in DMSO on the agar media for all bacterial strains. The antibiotic *Meropenem* and *Gentamycin* were used as standard drugs against Gram-negative bacteria. *Meropenem* and *Ampicillin* were used as standard drugs against Gram-positive bacteria. The plates inoculated with bacteria were incubated for 24 h at 37°C. After the period of incubation, the zone of inhibition produced by the test compounds was measured in distance (mm). The screening tests were performed in triplicate, and the results were taken as a mean of three determinations.

The tested samples diffuse through the agar around its disks and inhibit germination of the microorganism by characteristic zones of inhibition depending on the microorganism sensitivity to the test sample, then measuring the inhibition zones diameters in mm*.*

### Antifungal activity by PDA

The activity of the synthesised compounds on *F. oxysporum* isolate was carried out *in vitro* on PDA. A total of 39.0 g of PDA and 0.3 g of chloramphenicol were mixed and heated to obtain 1.0 l of standard medium. Flasks containing 100 ml PDA were autoclaved, then allowed to cool to 55–60°C. Appropriate volumes of synthesised compounds as stock solutions (10^4^ μg.ml^−1^, the active ingredient of each in DMSO) were added to the media to give a final concentration of 20, 40, 80, 120, and 160 μg.ml^−1^. Petri plates were inoculated in the centre with 5-mm mycelium disks of 5 days old culture *F. oxysporum* and incubated at 25°C. Fungal colonies’ diameters were measured after 24 and 72 h and the mycelium growth rate (MGR, cm^2^/day) was calculated by using the following equation:

$$MGR=\frac{{\left(d_{2}/2\right)}^{2}-{\left(d_{1}/2\right)}^{2}\times \pi }{t}$$

Where MGR represents mycelium growth rate, d_2_ is the average diameter of the colony in cm after 72 h, d_1_ is the average diameter of the colony after 24 h, t is the time of incubation [37]. The experimental design was a completely randomised design (CRD) with five replicates.

### Statistical analysis

All results are presented as the mean of triplicate ± standard deviation (SD) unless indicated otherwise. Paired sample *t* tests were performed, and the two-tailed significance (*P*-value) was determined, and *P*-value ≤0.05 was considered as statistical significance. Also, linear regression was performed and Pearson correlation coefficient was calculated.

## Results and discussion

Spectroscopic analysis of chalcone and pyrazole compounds was carried out by using different spectroscopic techniques including ^1^H NMR, ^13^C NMR, and FT-IR.

### ^1^H NMR of adamantyl chalcone

Figure 3A reveals the ¹H NMR of prepared adamantly chalcone. The peak appeared at δ 7.67 attributed to α proton in C-2 and at δ 7.75 to β proton in C-3. A collection of signals observed in the aromatic region δ 8.58–7.36 ppm is due to aromatic protons, the peaks at δ 2.75–1.90 ppm correspond to adamantyl group and at 4.9, 3.58 indicate the presence of ethanol.

### ^1^H NMR of pyrazole compounds

**Figure 4:**
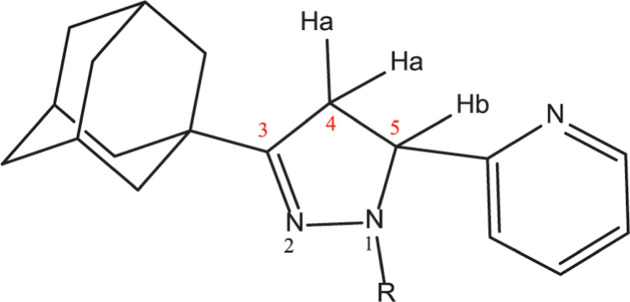
General scheme of pyrazoline compound

In ^1^H NMR spectra, the three protons (Ha and Hb) on pyrazoline structure are non-equivalent and therefore have different chemical shifts as shown on Figure 4. Ha and Hb attached to the C-4 and C-5 carbon atoms of the pyrazoline ring gave peak positions. The methylene protons of pyrazoline ring Ha appeared in the region approximately δ 2.870–3.340 ppm, and Hb appeared in the region δ 3.404–3.830 ppm, is the most de-shielded due to its close proximity to a pyridine ring. The peaks are observed for these three protons which supported the formation of pyrazole compounds. A signal at δ 2.026–1.691 ppm is assigned to adamantyl protons attached to pyrazoline ring at C-3. Moreover, a collection of signals observed in the aromatic region δ 8.809–7.122 ppm is due to aromatic protons at the first and fifth positions of the pyrazoline ring.

**Figure 5:**
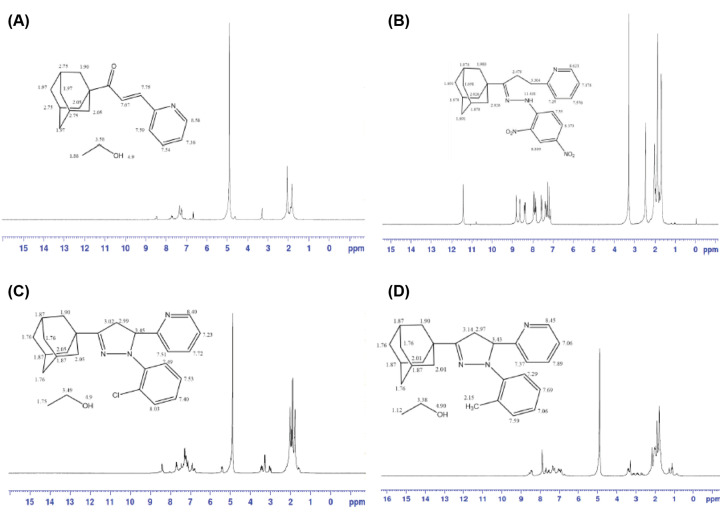
^1^H NMR of synthesised adamantyl chalcone and pyrazole compounds (**A**) ^1^H NMR of chalcone, (**B**–**D**) ^1^H NMR of RS-1, RS-3, and RS-5 respectively.

Figure 5B shows the ^1^H NMR spectrum of compound RS-1. The peak appeared at 2.47 indicate to ***Ha*** proton and at 3.304 to ***Hb*** proton. There is no double bond in carbons 4 and 5 because the spectrum appeared at a low chemical shift in high field region, where if there are double bond it should appear at downfield approximately at (7.56–7.12). The peak at δ 11.43 ppm for NH due to the ring open in position 1. Figure 5C shows the ^1^H NMR spectrum of ***RS-3*** chemical shifts. The methylene protons of pyrazoline ring ***Ha*** appeared in the region δ 3.02–2.99 ppm and ***Hb*** appeared in the region δ 3.45 ppm. A signal at δ 2.05–1.76 ppm is assigned to adamantyl protons attached to pyrazoline ring at C-3. In Figure 5D, the methylene protons of pyrazoline ring ***Ha*** appeared in the region δ 3.14–2.97 ppm and ***Hb*** appeared in the region δ 3.43 ppm. A signal at δ 2.01–1.76 ppm is assigned to adamantyl protons attached to pyrazoline ring at C-3.

### ^13^C NMR analysis of adamantyl chalcone

**Figure 6:**
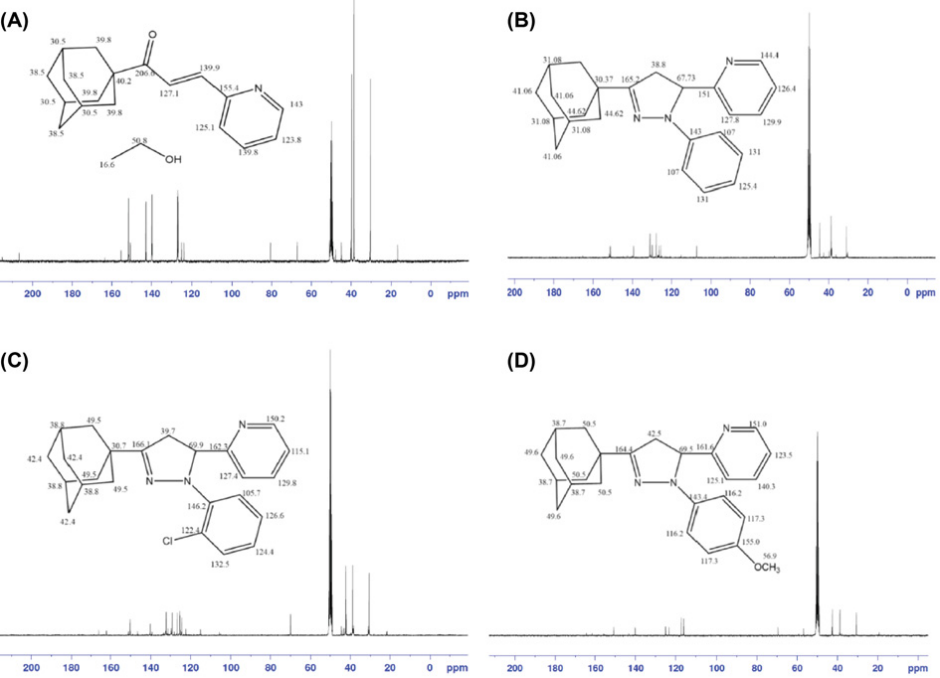
^13^C NMR of synthesised adamantyl chalcone and pyrazole compounds (**A**) ^13^C NMR of chalcone. (**B**–**D**) ^13^C NMR of RS-2, RS-3, and RS-4 respectively.

Figure 6A shows the ^13^C NMR spectrum of adamatyl chalcone. The peak appeared at δ 127.1 attributed to carbon 2 and at 139.9 to carbon 3. A collection of signals observed in the aromatic region δ 155.4–123.8 ppm are due to pyridine carbon, and the peaks at region δ 40.2–30.5 ppm correspond to adamantyl carbon.

### ^13^C NMR of *RS-2, RS-3*, and *RS-4*

Figure 6B reveals the ^13^C NMR spectrum of compound RS-2, a signal at δ 44.62–30.37 ppm is assigned to adamantyl carbon attached to azole ring at C-3. Two signals at δ 38.8 and δ 67.7 ppm are assigned to C-4 and C-5 respectively. One signal at δ 165.2 ppm is attributed to C-3 in the azole ring which is a carbon attached to electronegative nitrogen by a double bond is de-shielded due to its sp^2^ hybridisation and electronegativity of nitrogen. A collection of signals appeared in the region δ 144.4–107.0 ppm which are assigned to aryl carbons, the intense peak at δ 50.5 ppm back to carbon in ethanol. Figure 6C shows the chemical shifts of RS-3, a signal at δ 49.5–30.7 ppm is assigned to adamantyl carbons attached to azole ring at C-3. Two signals at δ 39.7 and 69.9 ppm are assigned to C-4 and C-5 respectively. One signal at δ 166.1 ppm is attributed to C-3. A collection of signals appeared in the region δ 150.2–105.7 ppm which are assigned to aryl carbons, the intense peak at δ 50.5 ppm back to carbon in ethanol. Figure 6D exhibits the chemical shifts of compound RS-4. A signal at δ 50.5–30.6 ppm is assigned to adamantyl carbon attached to azole ring at C-3. Two signals at δ 42.5 and δ 69.5 ppm are assigned to C-4 and C-5 respectively. One signal at δ 164.4 ppm is attributed to C-3 in the azole ring which is a carbon attached to electronegative nitrogen by a double bond is de-shielded due to its sp² hybridisation and electronegativity of nitrogen. A collection of signals appeared in the region δ 155.0–116.2 ppm which are assigned to aryl carbons. The methoxy peak appeared at 56.9 and the intense peak at δ 50.5 ppm back to carbon in ethanol.

### FT-IR of adamantyl chalcone

**Figure 7:**
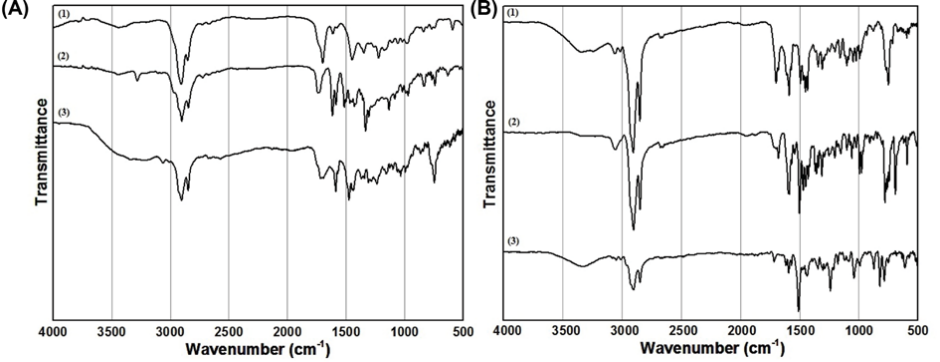
FT-IR spectra (**A**) adamantyl chalcone (1), RS-1 (2), and RS-3 (3). (**B**) RS-5 (1), RS-2 (2) and RS-4 (3)

In FT-IR spectrum of adamantyl chalcone (Figure 7A(1)) showed sharp peaks at 1716 cm^−1^ correspond to C = O, the peak appeared at 2904 cm^−1^ (C–H stretching of aliphatic adamantyl sp^3^ hybridisation), 3200 cm^−1^ (C–H stretching of aromatic ring sp^2^ hybridisation), 3393 cm^−1^ to O–H from ethanol, 1617 cm^−1^ back to C = C, C–C aromatic, 1223 cm^−1^ to C–H bend in pyridine and 1449 cm^−1^ to C = N.

### FT-IR spectroscopy of RS compounds

In FT-IR spectrum of compound RS-1 (Figure 7A(2)), the sharp peaks at 3286 and 2899 cm^−1^ C–H are corresponded to stretching of aromatic ring sp^2^ hybridisation C–H stretching of aliphatic adamantyl sp^3^ hybridisation respectively. The bands appeared at 3446 cm^−1^ confirm the presence of N–H functional group which indicate the ring open, the peaks appeared at 1737, 1616 cm^−1^ back to C = C, C–C aromatic, at 1447 cm^−1^ to C = N, 1134 cm^−1^ C–H bend, and at (1512, 1334 cm^−1^ N–O). The absorption bands due to the nitro group: N–O asymmetric stretch from 1512 cm^−1,^ N–O symmetric stretch from 1334 cm^−1^. They are at lower wavenumbers than usual (1550–1360 cm^−1^) because the nitro group is conjugated with the benzene ring. Figure 7A(3) showed peaks at 3066 cm^−1^ (C–H stretching of aromatic ring sp^2^ hybridisation), 2901 cm^−1^ (C–H stretching of aliphatic adamantyl sp^3^ hybridisation) for compound RS-3. The bands appeared at 1688, 1597 cm^−1^confirm the presence of C = C, C–C aromatic, at 1464 cm^−1^ to C = N and 1301 cm^−1^ to C–H bending and the peak at 740 cm^−1^ confirm the presence of C–Cl.

Figure 7B(1) showed peaks at 3052 cm^−1^ (C–H stretching of aromatic ring sp^2^ hybridisation), 2887 cm^−1^ (C–H stretching of aliphatic adamantly sp^2^ hybridisation) are corresponding to RS-5. The bands appeared at 1688 cm^−1^, 1583 cm^−1^ confirm the presence of C = C, C–C aromatic, at 1449 cm^−1^ to C = N and 1301 cm^−1^ to C–H bend. RS-2 showed sharp peaks at 3059 cm^−1^ (C–H stretching of aromatic ring sp² hybridisation), 2901 cm^−1^ (C–H stretching of aliphatic adamantyl sp^3^ hybridisation) as shown in Figure 7B(2), the bands appeared at 1675 cm^−1^, 1597 cm^−1^ confirm the presence of C = C, C–C aromatic, at 1491 cm^−1^ to C = N and 1307 cm^−1^ to C–H bend. In FT-IR spectrum of compound RS-4, the peaks at 3060 cm^−1^ (C–H stretching of aromatic ring sp² hybridisation), 2914 cm^−1^ (C–H stretching of aliphatic adamantyl sp^3^ hybridisation as shown in Figure 7B(3), the bands appeared at 1695 cm^−1^, 1597 cm^−1^ confirm the presence of C = C, C–C aromatic, at 1517 cm^−1^ to C = N and 1241 cm^−1^ to C–H bend, the peak at 3336 cm^−1^ due to OH in ethanol.

### TLC

TLC used to perform the purity of prepared compounds using ethyl acetate:hexane (2:5) as the mobile phase. The Retention Factor (*R_f_*) values for the starting material and the products are included in Table 1. The *R_f_* of adamantyl chalcone appeared at 0.54. All molecules had purity percentage more than 90%.

**Table 1:** *R_f_* values for the prepared compounds RS 1-5

Synthesised compounds	*R_f_*	Starting materials	*R_f_*
RS-1	0.62	B1	0.18
RS-2	0.60	B2	0.66
RS-3	0.58	B3	0.78
RS-4	0.38	B4	0.56
RS-5	0.56	B5	0.74

### Melting point and percentage yield

Table 2 includes some of the most important physicochemical properties; namely, the melting points, colour and percentage yield of RS compounds. First, 70–90% yield percentage was obtained from the reaction which is considered very high and reflects accurate and optimised conditions were maintained throughout the reaction. The colours of compounds are due to the presence of chromophore conjugation. It is a region in the molecule where the energy differs between two separate molecular orbitals and falls within the range of the visible spectrum. Visible light that hits the chromophore can thus be absorbed by exciting an electron from its ground state into an excited state. The electrons jump between energy levels that are extended pi orbitals, created by a series of alternating single and double bonds, in aromatic systems.

**Table 2:** Melting point, colour, and percentage yield of RS compounds

Synthesised compounds	Melting point (°C)	Colour	Present yield	Molecular formula	Molecular weight (g/mol)
Chalcone	95–97	Bright yellow	70.4%	C_15_H_12_O	208.26
RS-1	182–184	Orange	88.3%	C_24_H_25_N_5_O_4_	447.49
RS-2	107–109	Dark yellow	78.7%	C_24_H_26_N_3_	357.48
RS-3	Oily	Yellowish-brown	71.7%	C_24_H_26_N_3_CL	391.93
RS-4	82–83	Brownish-orange	75.1%	C_25_H_29_N_3_O	387.47
RS-5	70–72	Yellowish-orange	89.6%	C_25_H_29_N_3_	371.51

Molecular formula and molecular weight of RS compounds. The molecular formula and molecular weight for synthesised pyrazole compounds are also listed in the table.

### Antibacterial activity evaluation

The inhibition zone diameters (IZDs) of synthesised compounds against Gram-negative bacteria species and Gram-positive bacteria species are summarised in Table 3. The investigation of the antibacterial screening of the test samples ***RS 1-5*** revealed that all these compounds exhibited different degrees of antibacterial activity in relation to the tested microbial species and showed moderate to weak antibacterial activity against all organisms. The results of MICs revealed that the tested compounds could act as good antibacterial agents at higher concentrations, and no inhibition zone at a lower concentration. Adamantyl chalcone showed good to moderate activity against bacterial strain.

**Table 3:** The IZDs of the synthesised compounds RS 1-5 against bacterial species in millimetres

Microorganism						
Antibiotic	*Bacillus subtilis*	*Staphylococcus aureus*	*Pseudomonas aeruginosa*	*Klebsiella pneumonia*	*Salmonella typhimurium*	*Escherichia coli*
	Gram +	Gram +	Gram −	Gram −	Gram −	Gram −
Standard	Meropenem	Ampicillin	Meropenem	Meropenem	Meropenem	Gentamycin
	30	30	25	25	20	20
Adamantyl chalcone	19.5	13.7	12.7	17.7	9.3	11.3
RS-1	8.7	9.2	13.3	15.3	10.3	10.7
RS-2	11.3	11.7	9.7	12.3	9.3	11.3
RS-3	9.3	10.7	8.7	10.3	8.3	11.3
RS-4	13.7	11.7	8.7	13.3	9.3	10.3
RS-5	12.3	10.7	11.7	12.7	10.7	10.7

*Data are expressed as a mean of three determinations (*n*=3).

### Antifungal activity evaluation

In Table 4, the synthesised compounds revealed that all compounds reduced the mycelial growth rate of *F. oxysporum* fungus at concentrations (20–160 μg/ml) and clearly showed antifungal activity*.* The fungal growth reduction induced by the compounds RS-1, RS-2, RS-3, RS-4 and RS-5 at 160 μg/ml were 6, 31, 54, 49, and 56%, respectively, compared with control. Besides, the reductions were highly negatively correlated with highly correlation coefficient, as shown in Table 5. The compounds RS-3, RS-4, RS-5 were highly active against *F. oxysporum*, which may be attributed to the presence of an electron-donating group, chlorine in the *ortho* position, methoxy in *para* position and methyl in the *ortho* position of the benzene ring, respectively. The test samples RS-2 has shown moderate activity against *F. oxysporum*, which may be due to no substituents on the benzene ring, while RS-1 marked the lowest activity against the fungus, which may be due to the presence of electron-withdrawing –NO_2_ groups on the aromatic benzene ring [38]*.* The adamantyl chalcone showed good activity against *F. oxysporum* fungus (66%).

**Table 4:** Effect of prepared compounds RS 1-5 against the MGR (cm²/day) of the fungus *F. oxysporum* grown on PDA medium amended with the compounds at concentrations (0–160 μg/ml) and incubated at 25°C

Concentration (μg/ml)						
Compound	0	20	40	80	120	160
Adamantyl chalcone	7.8	4.9	4.5	3.7	3.1	2.5
RS-1	7.8	7.6	7.6	7.4	7.4	7.3
RS-2	7.8	5.9	5.7	5.6	5.5	5.4
RS-3	7.8	4.6	4.3	4	3.9	3.6
RS-4	7.8	5.3	4.9	4.8	4.4	4
RS-5	7.8	4.5	4.4	4.2	3.8	3.4

*Data are expressed as a mean of five determinations (*n*=5).

**Table 5:** The linear regression and correlation coefficient of the MGR induced by the prepared compounds at concentrations 0–160 μg/ml

Compound	Linear regression equation	Correlation coefficient (R^2^)
Chalcone	Y = –0.9343x + 7.6867	0.8675
RS-1	Y = –0.0943x + 7.8467	0.9242
RS-2	Y = –0.38x + 7.3133	0.6151
RS-3	Y = –0.6686x + 7.04	0.6454
RS-4	Y = –0.6229x + 7.38	0.7461
RS-5	Y = –0.6943x + 7.1133	0.6755

The test compounds reduced significantly the MGR of *F. oxysporum* correlated with concentration with highly correlation coefficient, summarised in Table 5. A close investigation of the *in vitro* antifungal activity profile of the trisubstituted pyrazolines gives a clear picture of the structural activity correlations among the compounds (RS 1-5) under study.

## Conclusion

In the present study, five different pyrazole adamantyl chalcone molecules were developed and characterised. Both physical and chemical properties were investigated to find out their melting points, appearance, colour, chemical structure, and purity by utilising NMR, FT-IR, and TLC. Also, their antibacterial and antifungal properties were well investigated. To our knowledge, this is the first work that develops pyrazole and pyrazoline-based adamantyl chalcone and investigates them. Our study concludes that the heterocyclic adamantyl chalcones developed showed moderate antibacterial effects, and their effect was dose-dependent. Thus, increasing the dose of the used molecules enhanced their antibacterial effects significantly. Also, the molecules RS 3, 4, and 5 showed very high antifungal activity, especially when at high doses. However, RS1 and 2 had a modest to average antifungal activities in comparison with other molecules. In conclusion, adamantyl heterocyclic chalcones have promising pharmacological effects and may form a superior alternative to the classical chalcones due to the high lipophilicity they have, which may enhance their pharmacokinetic properties.

## Abbreviations

FTIR: fourier-transform infrared
HOAc: acetic acid
MGR: mycelium growth rate
MHA: Mueller–Hinton agar
MIC: minimum inhibitory concentration
PDA: potato dextrose agar
ppm: parts per million
*R_f_*: retention factor
RS: chalcone molecules code
TLC: thin-layer chromatography
TMS: tetramethylsilane
